# Endovenous Laser Ablation-Phlebectomy-Sclerotherapy Hybrid Treatment for Venous Insufficiency: A Case Report

**DOI:** 10.7759/cureus.42161

**Published:** 2023-07-19

**Authors:** Anjani H Turaga

**Affiliations:** 1 Medicine and Surgery, Gandhi Medical College, Hyderabad, IND

**Keywords:** foam sclerotherapy, sclerotherapy, phlebectomy, varicosities, varicose veins, venous insufficiency, evla

## Abstract

A hybrid treatment approach was used to successfully manage bilateral varicose veins in a 50-year-old female patient. The patient had venous insufficiency and presented with pain, swelling, and discoloration in her legs. The treatment plan consisted of two sessions of endovenous laser ablation (EVLA) to treat venous reflux, followed by two phlebectomies for residual bulging varicose veins. After a three-month follow-up, an ultrasound evaluation showed no venous reflux, indicating successful treatment. However, small reticular and spider veins remained, so the patient underwent four sessions of foam sclerotherapy using polidocanol as the sclerosant. On her three-month follow-up post-sclerotherapy, the patient reported significant improvement in symptoms, and ultrasound evaluation showed no venous reflux in major veins. The hybrid approach combining EVLA, sclerotherapy, and phlebectomy provided effective treatment for the patient, resulting in symptom improvement and positive cosmetic outcomes.

## Introduction

Venous insufficiency is a common condition that affects numerous people worldwide. It is characterized by the inability of the veins to adequately circulate blood, leading to the development of varicose veins and other related symptoms. Various treatment options have been developed to manage venous insufficiency, including endovenous laser ablation (EVLA), phlebectomy, and sclerotherapy.

EVLA is a minimally invasive procedure that involves the use of laser energy to seal damaged veins, thereby redirecting blood flow to healthier veins. The laser used is guided by ultrasound imaging, ensuring that it targets the correct area. Every patient is anesthetized using tumescence before the procedure to minimize discomfort during the process. Tumescence also helps protect the surrounding tissues from laser damage. A recent study by Chen et al. [1] showed that tumescent-assisted EVLA was an effective and safe method for treating superficial venous insufficiency (SVI).

Phlebectomy is another treatment option that involves the removal of small veins on the surface of the skin. This is done by making small punctures using a needle, and the veins are then removed. This technique is particularly useful for veins that are too small to be treated using EVLA or sclerotherapy. Bulging veins may also be removed using this technique, especially if they are causing significant discomfort to the patient [2].

Sclerotherapy involves the injection of a solution into damaged veins, causing them to close and redirecting blood flow to healthier veins. This solution is usually a type of saline solution, foam, or chemical sclerosant that irritates the lining of the vein and causes it to swell shut, eventually leading to its disappearance. The sclerosants used are chosen based on the type of vein being treated and the severity of venous insufficiency. A recent study by Star et al. [3] suggests that sclerotherapy using polidocanol foam was an ideal sclerosant for treating venous insufficiency.

It is also worth noting that different treatment options can be used in combination to achieve better outcomes. For example, in patients with combined superficial and deep vein insufficiency, ablation of reflux in the superficial venous system may lead to the abolishment of deep vein reflux, making foam sclerotherapy a good treatment option for these patients. A study by Watanabe et al. [4] also suggests that the simultaneous use of EVLA and sclerotherapy can lead to a more significant reduction in symptoms and an improved quality of life for patients, resulting in fewer second-stage interventions such as vein stripping.

In the treatment of venous insufficiency, several different options are available depending on the severity and type of venous disease. These options include EVLA, radiofrequency ablation, ultrasound-guided foam sclerotherapy, phlebectomy, and the combined use of different treatment options.

## Case presentation

This case involves a 50-year-old female patient who presented with symptoms of venous insufficiency, including leg pain, swelling, and varicose veins. Her primary care physician diagnosed her with varicose veins bilaterally on both lower extremities six years ago.

An ultrasound examination revealed reflux in the great saphenous vein on the left lower extremity, the short saphenous vein on the right lower extremity, and incompetent perforator veins bilaterally. She was classified as a CEAP class 3 patient. Given the severity of her condition, a combination of EVLA and foam sclerotherapy was considered the most appropriate treatment option. Phlebectomy was also planned to remove the visible varicosities.

The first EVLA procedure was performed on her right lower extremity to treat the short saphenous vein reflux, using ultrasound-guided ablation. Tumescent anesthesia was administered, and the laser was used to ablate a 38 cm segment of the vein. The laser was used for 139 s, delivering 977.9 J of energy. After the procedure, the patient was prescribed compression stockings and given instructions on how to care for the treated area. A follow-up ultrasound confirmed the absence of reflux in the treated vein, indicating a successful procedure.

The second EVLA procedure targeted the great saphenous vein reflux on her left lower extremity. Tumescent anesthesia was administered, and a 40 cm segment of the vein was ablated using the laser for 77 s, delivering 541.6 J of energy. After the procedure, a follow-up ultrasound was scheduled for one week later to confirm the absence of reflux, which was successfully achieved.

**Figure 1:**
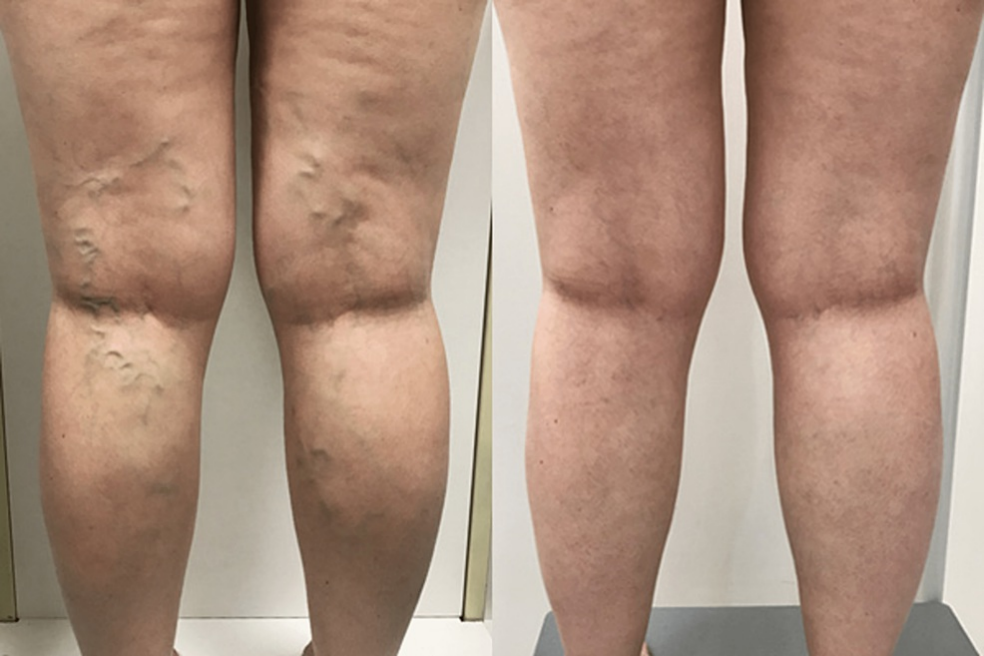
Patient’s lower limbs prior to treatment (left) and post-EVLA (right) EVLA, endovenous laser ablation

During the three-month healing period, the patient wore compression stockings to facilitate recovery and minimize complications. At the follow-up appointment, the patient reported significant symptom improvement and expressed satisfaction with the treatment outcome. The follow-up ultrasound showed no reflux in both the short saphenous vein and the great saphenous vein, indicating successful obliteration of the abnormal veins. A comparative picture of the patient’s legs prior to treatment and at her follow-up appointment post-EVLA is shown in Figure 1.

A phlebectomy was performed on the patient’s left lower extremity three months later to treat surface varicosities in the calf region. Fifteen incisions were made, resulting in a blood loss of approximately 20 mL. The patient received post-phlebectomy instructions, including wearing compression stockings for a week and avoiding activities such as heavy lifting, prolonged standing or walking, and hot baths or saunas.

A week after the left lower extremity phlebectomy, a phlebectomy procedure was scheduled for the patient’s right lower extremity in the calf region to treat surface varicosities. Fourteen incisions were made, and the procedure resulted in a blood loss of approximately 25 mL. Post-procedure, the patient was instructed on the proper application of compression bandages to the lower extremities.

A follow-up ultrasound was conducted six months later, revealing a remarkable improvement in visible varicosities. The treatment plan, consisting of two EVLA procedures followed by subsequent phlebectomies in both lower extremities, was considered successful in eliminating abnormal veins and reducing varicosities.

Persistent small spider and reticular veins were managed through four foam sclerotherapy sessions, with two sessions on each leg and one-week intervals between sessions. Polidocanol was the sclerosant of choice, injected directly into the targeted small veins using a fine needle. The patient reported minimal discomfort during the procedure, and post-treatment instructions included wearing compression stockings for a week and avoiding prolonged standing or walking, as well as hot baths or saunas.

**Figure 2:**
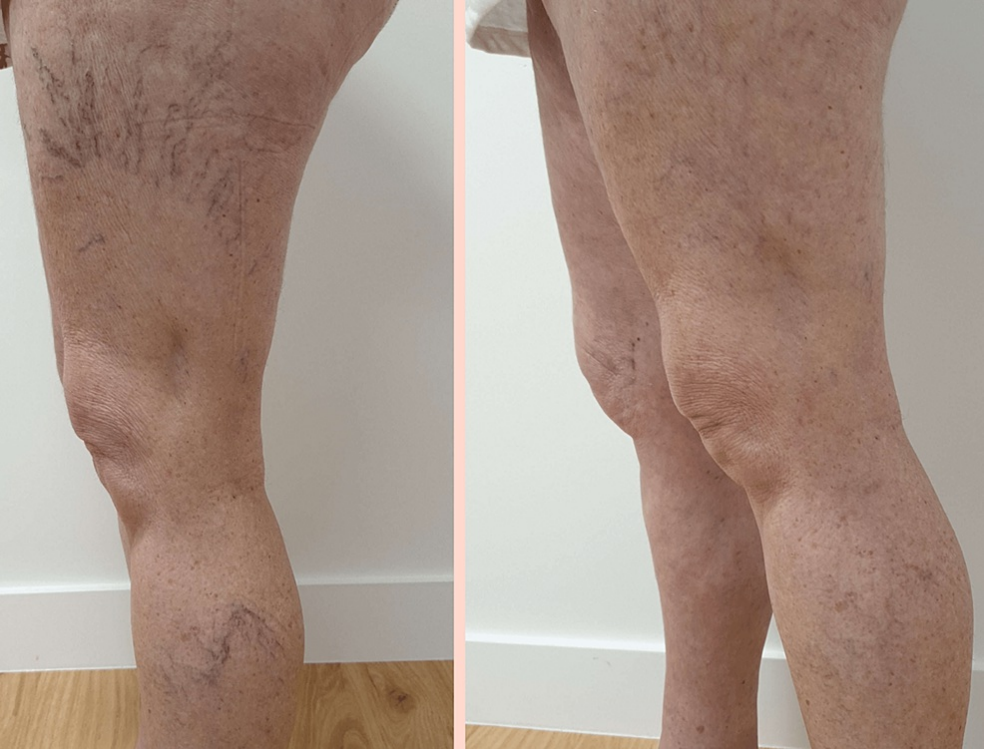
Patient’s lower limb prior to sclerotherapy (left) and after sclerotherapy sessions (right)

A three-month follow-up appointment was scheduled, including an ultrasound examination. The ultrasound showed no varicosities, and upon examination, no spider or reticular veins were visible. The patient was advised to continue wearing compression stockings regularly and to schedule a follow-up appointment in six months. A comparison between the patient’s limbs prior to sclerotherapy and post the sessions is shown in Figure 2.

## Discussion

This case details a successful treatment plan for chronic venous disease with varicose veins using minimally invasive methods such as EVLA, phlebectomy procedures, and foam sclerotherapy. A study published by Quarto et al. [5], which compared EVLA and surgery for the treatment of varicose veins, found that EVLA was a safe and viable alternative to surgery. Similar findings were observed in this case.

Furthermore, the use of ligation and vein stripping as primary treatments for SVI and varicose veins has decreased due to the effectiveness of newer minimally invasive treatments like EVLA and foam sclerotherapy. Similar research findings were discussed in a study by Liu et al. [6], which compared radiofrequency ablation and foam sclerotherapy as alternative methods to surgery for treating varicose veins. Our case report highlights the success of a chronic case without the need for major surgeries.

A combination of the three treatments proved to be more effective for the patient, as detailed above, compared to a singular varicose vein treatment. This is further supported by a study conducted by Hauzer et al. [7], which compared the effectiveness of different minimally invasive techniques for treating varicose veins and showed improved patient outcomes when multiple hybrid treatments were used in combination.

A paper by Balint et al. [8] mentions the lack of randomized controlled trial (RCT) evidence on the effectiveness of EVLA and foam sclerotherapy for treating venous insufficiencies. Our case report contributes to the body of evidence suggesting that these treatments can be effective in such cases.

## Conclusions

This case not only highlights the effectiveness of minimally invasive treatments, such as EVLA, phlebectomy procedures, and foam sclerotherapy, for the treatment of chronic venous disease with varicose veins but also emphasizes the importance of a comprehensive and individualized treatment plan. Moreover, the combination of multiple treatment options can provide better outcomes for patients when treating varicose veins. Patient compliance and follow-up care are crucial for ensuring the success of these treatments, as well as preventing complications and recurrence.
